# Developing a Tool for Assessing the Process of Seeking Health Information: Online Think-Aloud Method

**DOI:** 10.3390/healthcare12101039

**Published:** 2024-05-17

**Authors:** Asim Alhejaili, Heather Wharrad, Richard Windle

**Affiliations:** 1 School of Health Sciences, University of Nottingham, Nottingham NG7 2HA, UK; heather.wharrad@nottingham.ac.uk (H.W.); richard.windle@nottingham.ac.uk (R.W.); 2 College of Nursing, Taibah University, Medina 42353, Saudi Arabia

**Keywords:** nursing students, performance tool, seeking health information, clinical practice

## Abstract

Nursing students can access massive amounts of online health data to drive cutting-edge evidence-based practice in clinical placement, to bridge the theory–practice gap. This activity requires investigation to identify the strategies nursing students apply to evaluate online health information. Online Think-Aloud sessions enabled 14 participants to express their cognitive processes in navigating various educational resources, including online journals and databases, and determining the reliability of sources, indicating their strategies for information-seeking, which helped to create this scoring system. Easy access and user convenience were clearly the instrumental factors in this behavior, which has troubling implications for the lack of use of higher-quality resources (e.g., from peer-reviewed academic journals). The identified challenges encountered during resource access included limited skills in the critical evaluation of information credibility and reliability, signaling a requirement for improved information literacy skills. Participants acknowledged the importance of evidence-based, high-quality information, but faced numerous barriers, such as restricted access to professional and specialty databases, and a lack of academic skills training. This paper develops and critiques a Performative Tool for assessing the process of seeking health information using an online Think-Aloud method, and explores factors and strategies contributing to evidence-based health information access and utilization in clinical practice, aiming to provide insight into individuals’ information-seeking behaviors in online health contexts.

## 1. Introduction

Various national and international recommendations have been formulated in relation to obtaining and assessing evidence-based health information (EBHI), all of which stress that good performance in terms of information-seeking behaviors (ISBs) can provide self-directed skills to filter resources while looking for health information, in order to avoid any uncertainties or risks that might otherwise be inherent in such information [1,2,3]. Extensive research has shown that the ability to use critical thinking among nursing interns while seeking health information is limited. Along with difficulties in other aspects such as analyzing complex patient situations and the ability to predict patient needs and potential outcomes, newly graduated nurses are dependent on help and guidance from experienced nurses [4,5,6,7]. In line with the general need for more insight into the theory–practice gap and other issues in the transition to clinical practice, more research is needed to explore and enhance ISBs and critical thinking dispositions among newly qualified nurses, which are directly rooted in their experiences from internship programs [4]. Thus, this study explores some of these important challenges and considers them in the context of the training of nursing intern students (NIS) for practice. No previous studies were found exploring ISBs among NISs in Saudi settings.

There are several tools available to assess nursing students’ performance in a specific task in clinical practice, as affirmed by numerous nursing studies [8,9,10]. One commonly used tool to assess knowledge and skills for practice is the National Council Licensure Examination for Registered Nurse (NCLEX-RN) [11]. Another tool is using simulation labs to allow direct observation of nursing students’ performance and ability to make clinical decisions in controlled environments. In such assessments, students are given scenarios and asked to deliver care based on the checklist according to the local standard of care [12]. In addition, clinical instructors can provide a performance evaluation for their students after observing and monitoring them during their clinical rotations. This method of evaluation can provide a valuable insight to assess students’ performance and progression in practice [13]. Students’ performance can also be assayed through self-assessment, whereby students can evaluate their own knowledge or skills and their ability to apply them in practice [14]. These methods of evaluation can provide comprehensive insights into students’ strengths and weaknesses in practice. While NISs may have engaged in ISBs during clinical placements, there are no tools available to assess their performance in seeking health information during the transition to clinical practice. Therefore, the focus of the study was on the process of seeking health information for clinical practice application. ISBs are the activities of a person who can recognize their information needs, searching for such information in any way and seeking to use or transfer such information [15]. Many studies about ISBs in health have concluded that finding and using information is defined as the process of identifying health information needs (HINs), and locating, collecting, reviewing and using the retrieved information to obtain information based on evidence [16,17,18,19,20].

This study differs from existing research by exploring ISBs among nursing students in Saudi clinical settings, offering an exploration of the unique aspects within the Saudi healthcare environment. Unlike previous research that may have provided more generalized insights, such as recruiting participants who were not working in clinical practice [21,22,23,24,25], other studies have focused on different settings and care pathway levels, such as physicians [26,27,28,29]. This study delves into the specific practical challenges and strategies applied by NISs in this characteristic context. The multifaceted exploration encompasses aspects such as cognitive process, practical research skills, and healthcare infrastructure, which collectively shape the information-seeking strategies of NISs.

By providing a detailed account of these contextual details, this study holds the potential to inform targeted improvements in internship program and educational practices within the concept of EBHI. It also may uncover common patterns and variations in methods of seeking health information among NISs across diverse cultural and clinical placement contexts. In recent years, there has been a growing emphasis on promoting the use of EBHI and enhancing health literacy [15,30]. There is a significant correlation between nursing students’ ISBs and their preference for EBHI. Nursing students who actively seek out evidence-based sources demonstrate a commitment to incorporating the best available research into their practice, thereby laying the groundwork for delivering high-quality patient care [31]. However, the quality of the information varied massively, which made it a challenge for students to distinguish between EBHI and misinformation in clinical practice [32].

Several factors emerged from the Think-Aloud method (TA) that allow the assessment of students’ skill and performance in seeking quality health information; thus, we decided to use these factors to create an innovative tool to assess their performance. TA as a qualitative data-collection method has been widely utilized in cognitive psychology as a means of gathering verbalizations regarding productive thinking, and as a means of understanding the development of thought in individuals [33,34]. The main aim of this phase was to develop a tool that could be used by students and educators to gauge their understanding and skill in seeking health information. Moreover, this tool is intended to achieve different outcomes, such as enhancing critical thinking and increasing their independence in practice. It is really important to approach this tool in a systematic process, based on an evidence-based model, to allow any adjustments to be made before full implementation [35]. Therefore, this stage of the research focused on the development of the Performance Tool (PT); a new measure for evaluating the skills of seeking EBHI among nursing students. In particular, it was intended to develop a practical tool that may help in evaluating the quality of health information assessment, and provide a means for seeking health information in practice, along with the possibility of exploring this area with a larger population of participants in similar or different contexts. The aims of this paper are thus to: (1) develop and critique the Performative Tool (PT), a novel approach for assessing the process of seeking health information using an online Think-Aloud method (TA); and (2) explore the factors and strategies contributing to the ability to access and utilize EBHI in clinical practice. The study thereby integrates the development and evaluation of the PT with an exploration of broader contextual factors of evidence-based practice (EBP).

## 2. Materials and Methods

### 2.1. Study Design

Online “Think-Aloud” was selected as a method to investigate 14 NISs’ information-seeking strategies through direct observation. It was chosen due to its advantages in capturing real-time cognitive processes while participants engaged in health information-seeking activities online [36]. This method allows for naturalistic observation in a familiar online environment, potentially reducing the impact of researcher presence on participant behavior and facilitating more authentic responses [25]. Additionally, the online format offers convenience and flexibility for both participants and researchers, allowing for greater reach and accessibility across diverse populations [37,38].

The proposed task was to provide participants with a scenario that represents a patient that they might come across in their clinical practice. The researchers adopted the idea of a problem-solving task through a clinical scenario to motivate participants to explore their ISPs with objectives that closely align with clinical practice. A clinical statement task was designed to encourage the use of diverse health information resources and evaluative strategies when seeking health information, and to utilize clinical reasoning. Therefore, the researchers used this task based on the Standard Saudi Care of Nursing Internship Program to encourage participants’ navigation of clinical information. At this juncture, the researchers needed to consider the clinical statements of the actual stage based on expert opinions. The experts were two clinical instructors who provide direct supervision to NISs in clinical placements for training purposes, such as education and teaching nursing skills. They were asked to evaluate all the statements in terms of readability and suitability for NISs. Clinical instructors evaluated these statements based on predefined criteria, including clinical relevance, accuracy, and alignment with nursing practice, to determine their suitability for the task. The clinical statements were employed in the TA to stimulate engagement and facilitate the extraction of criteria pertinent to the skills and strategies involved in seeking health information. Table 1 shows the eight clinical statements and their focus.

This study utilized convenience sampling, which is a common method in qualitative research. Non-random purposeful sampling does not require a specific number of participants that researchers have to comb through (in order to find detail-rich cases to use) [21,22]. In qualitative research, convenience sampling is a typical technique for selecting participants based on their availability and accessibility to researchers, who decided what information was applicable, and selected people willing to provide information based on their knowledge and experience [23]. Therefore, the sampling method was based on the intention or purpose of the study (i.e., to understand the thought process of the NISs who were interested in seeking health information in clinical placement), and then the selection criteria were allocated to the internship program.

Inclusion criteria were calibrated to recruiting a sample of nursing students in possession of a laptop with camera and audio input and screen-share capability. They completed all the educational requirements at Taibah University. At the time of recruitment, an invitation letter was sent to 30 nursing interns who had begun the program. Fourteen participants (P14) volunteered to complete the TA online task. The participants were requested to take part in a TA session where they were given a task and asked to talk through where they would seek information to understand and act on this clinical statement. PowerPoint was used to present the clinical statements, and Microsoft Teams was used to contact the participants. The sample size in TA typically ranges from 8 to 50 participants, with smaller numbers providing richer evidence on cognitive and decision processes [39,40,41,42].

**Figure 1:**
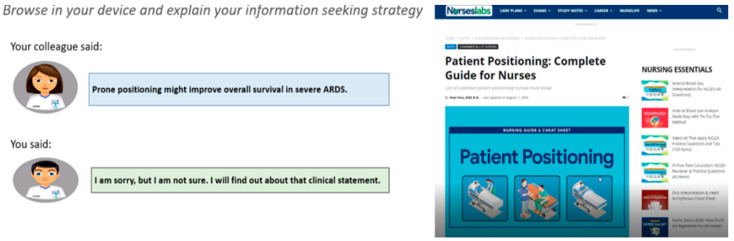
TA session.

Participants were given instructions and a brief description of the TA technique. For each clinical statement, a PowerPoint slide popped up to show the new clinical statements with instructions to guide them through a task. Participants chose which resources of information to see first and how. The statement represented a typical nursing care encounter, with decision-making focused on a request for formulating a clinical decision for a patient. After that, we traced NIS patterns and the strategies used in seeking health information to determine their interest in specific interactions, and whether they were following a proper ISP. Figure 1 shows an example clinical statement and how a participant interacted during the Thinking-Aloud online session.

### 2.2. Development of the Performance Tool (PT)

Researchers acknowledged that the TA method has been successful in providing an understanding of participants’ skills and behaviors when completing tasks about health-related information [39,43,44]. Several studies have highlighted that using TA to develop a tool that would support the understanding of the complex web-based strategies of seeking health information [45,46]. Thus, these factors were used to create a scoring system that could be used to evaluate the quality of the ISP in terms of obtaining EBHI, thereby allowing a more objective metric for measuring performance. Integrating the PT to assess NISs regarding seeking health information can be a valuable process for obtaining EBHI in practice. A well-designed tool can help NISs to acknowledge their own strengths and weaknesses, consequently helping to enhance EBP and decision-making, and no pre-existing tool for this purpose was found in the literature search. Performance was scored using the tool based on NISs’ responses to clinical statements in TA sessions using resources in different ways to find and evaluate the information to meet their needs.

This scoring system is derived from the data. The first step in this process was undertaken after collecting TA data. Three sources—participants’ thoughts, screen sharing activities, and the researcher’s notes—provided the data used to create the scoring system. The researchers employed a structured framework for note-taking during the TA sessions, focusing on capturing participants’ thought processes, information-seeking strategies, and challenges encountered during the task, thereby ensuring systematic data collection and analysis. All of the tool’s items were reviewed, revised, and resolved with the research team, who shared their insights, reviewed the features and functionality, and gave their feedback on its usability. The research team comprised individuals with expertise in nursing education, EBP, and research methodology, who utilized their collective knowledge to assess the usability of the tool, providing insights from various perspectives to ensure its effectiveness in evaluating the skills of seeking EBHI among nursing students. This process helped us refine the concept, make improvements, and facilitate the adoption of the scoring tool. Finally, Cohen’s Kappa was used for reliability to show how high the agreement was between two raters [47]; the value of 0.74 was “substantial” for this tool.

An overall ranking was performed based on 17 questions with yes or no answers (Table 2). Table 3 presents an example of using the tool to record the performance of an NIS to obtain EBHI for clinical statement number one, to illustrate part of the performance score. This tool can be used to explore the relationship between performance and other independent variables, such as information quality assessment and clinical statement level, to further analyze information-seeking skills. These relationships were analyzed using SPSS to extract the median, minimum, and maximum values, as well as to create a boxplot. The median is a measure of central tendency, which is used instead of the mean if the normality of the data distribution is not known (hard to verify in this study with only fourteen participants) [48]; the median identifies the typical and outlying responses in a data set. Due to the distribution of multiple datasets, boxplots were used to present the data graphically. They were useful for identifying similarities and differences between datasets.

## 3. Results

### 3.1. Assessment Criteria (AC) Scores

The data collected reveal that all criteria scored ranged from 0 to 8. Table 4 shows the assessment criteria score. A descriptive approach was used to analyze all assessment criteria data. The median score was relatively low [3], affected by some criteria with egregiously lower (outlying) scores, which influenced the overall results.

The highest median in the tool was 8 for relevance (AC4) and satisfaction (AC17), meaning that the NISs were able to find health information to address their needs for almost all clinical statements and were satisfied. This was followed by using keywords (AC2), which scored 7, related to using different keywords to locate the health information. Similarly, interpretation scored 7, whereby NISs were able to understand the clinical statements without asking the researchers for clarification (AC1), which scored 7.

**Figure 2:**
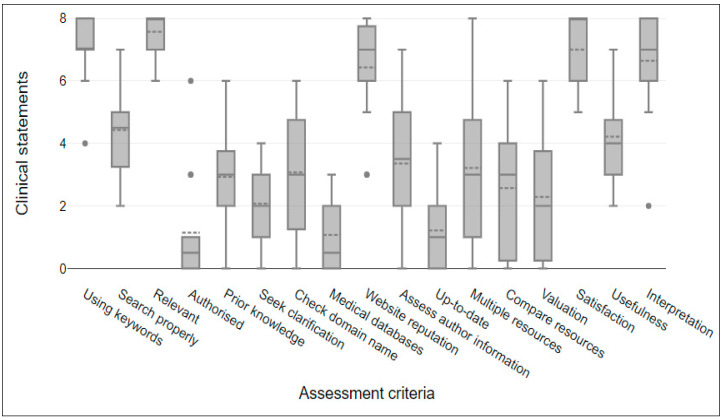
Assessment criteria scores for clinical statements.

Search engines properly (AC3) scored 4.5, referring to the ability to transform a clinical statement or specific HINs into a searchable sentence. The lowest score was 0.5 for AC9, which shows that databases (e.g., PubMed and MEDLINE) are unlikely to be used for obtaining clinical answers among NISs. Also, using professional organization websites (AC5) such as NICE, NHS, WHO, or MoH, scored 0.5. Furthermore, prior knowledge (AC6) and usefulness (AC13) scored 3 and 4, respectively. Figure 2 shows these statistics represented in a box plot to illustrate the variation for each assessment criteria in all clinical statements. The box plot shows the distribution of assessment criteria scores for clinical statements. The line inside each box shows the median number, and each box shows the interquartile range (IQR). The whiskers show the range, that is, the lowest to highest scores; the outliers are shown by single data points outside.

### 3.2. Clinical Statements Scores

The clinical statements (CSs) were about different subjects, such as medical diagnosis, contraindications, nursing routine care, guidelines, pain management, nursing responsibility, and patient education. Each NIS was recorded and assessed in relation to eight clinical statements (thus, each clinical statement level might score from 0 to 17 for each participant). The data collected reveal that all clinical statements scored ranged from 4 to 13. Table 5 shows the clinical statement scores in order to show the level of engagement. The statistical analysis of the clinical statement level was pulled from the assessment criteria tool. The median score was slightly lower at 8; clinical statements scores ranged between 7 and 9.

**Figure 3:**
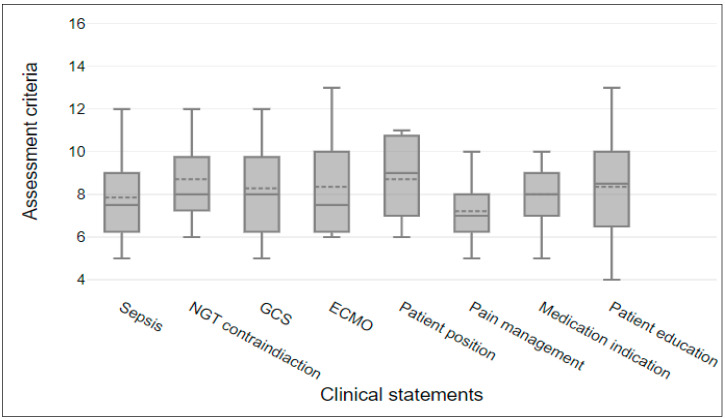
Clinical statements’ scores represented by box plots to show variation.

The highest clinical statement score was 9 for CS5, in which the NISs sought relevant information regarding patient position in ARDS. The second highest score was 8.5 for CS8, related to patient education related to method of breathing, followed by scores of 8 for CS2, CS3, and CS7, pertaining to NISs paying attention to nasogastric tube contraindication, GCS, and medication indication (respectively). The lowest score was 7 for CS6, related to pain management. Figure 3 shows the clinical statements’ variation in scores based on the participants’ scores. Across all the scenarios, the scores range from 4 to 13. The box plot shows the distribution of clinical statement scores for assessment criteria. The line inside each box shows the median number, and each box shows the interquartile range (IQR). The whiskers show the range, that is, the lowest to highest scores.

## 4. Discussion

This section discusses the results obtained from the PT related to information quality assessment and TA activity. It provides both an analytical and performance rating tool that has the potential for practical application in assessing information-seeking skills. This tool will help to evaluate NISs’ ISP in clinical practice to ensure that they have the necessary skills to obtain EBHI in practice. This discussion demonstrates two main areas of focus concerning NISs’ quality assessment, as applied to (1) the ISP and (2) the clinical statements.

### 4.1. Quality Assessment Applied by NISs

One significant finding from our study relates to the interpretation of the clinical question or statements, which are an essential part of clinical practice, as they help NISs to develop critical thinking skills and provide EBP [49]. Several reports have shown that understanding the nature of the question being asked and knowing the key component of the question would help to understand the question and develop a focused search strategy [50,51]. A study explored 114 nursing students’ abilities to identify the key component of 10 clinical questions and seek EBHI resources. The results presented in this study show that nursing students scored moderately in terms of their ability to understand and answer the clinical questions, with a mean of 5.5 out of 10; however, the median of 7 indicates that some egregiously low scores seriously reduced the mean value [52]. Consequently, the results imply that most participants typically had the ability to understand almost all CSs, as the motivation for their initiating the process of seeking health information.

Seeking clarification can help NISs to improve their ISP [53,54]. A study mentioned that nursing students might not seek clarification because they do not want to appear incompetent in front of their clinical instructors or patients [55]. In this study, the “seeking for clarification” criterion had a median of 2, indicating that participants were less likely to use a translator or to ask the researchers, and suggesting the possibility that they simply identified unknown words and ignored them. In accordance with the present results, a previous study has demonstrated that some healthcare professionals prefer to ignore unknown diseases, abbreviations, or medical terminology while seeking health information during practice [56].

Using keywords is an important strategy that NISs can use to obtain EBHI, such as words that describe the information being searched [57,58]. NISs can use keywords to narrow down their seeking process and obtain the most relevant health information more quickly [59]. For example, participants in this study used “heart failure”, “asthma”, “patient education” or “WHO guidelines”. Using keywords had a median score of 7, indicating that participants typically had the ability to use keywords in seven CSs out of eight for locating online health information. A survey that included 111 healthcare providers found that 94% had recently searched with keywords, and mentioned that a very low use of keywords is likely to result in not accessing the best quality evidence for EBP [60].

Searchable sentences refer to complete sentences that have the key elements for the clinical statements and are used to seek relevant health information [61]. The ability to transform a clinical statement or a need for health information into a searchable sentence was one of the challenges for NISs in finding EBHI. Using searchable sentences had a median score of 4.5, which means that participants typically had some issues with the ability to type into the search engine properly. However, “finding relevant health information” had a median score of 8, indicating that participants typically had the ability to find health information that was relevant to addressing their needs. A similar study assessed the awareness of appropriate health resources among the medical students at the University of Aligarh, and discovered that 71.81% of the trustworthy resources they used were relevant to their subject field [62]. When it comes to healthcare, where accurate information is of the utmost importance, the reliability of the resource is a key factor in figuring out how good an NISs’ ISP is [29].

Understanding how healthcare professionals interpret clinical questions, seek clarification, use keywords, and search properly is vital in achieving the objectives of this research. By exploring their interpretation and approach to these questions within the context of information-seeking behaviors, we can uncover valuable insights into the cognitive processes and decision-making strategies used in clinical practice. We discovered that nursing students emphasized the necessity of accessing accurate, reliable, and up-to-date health information to provide the best possible healthcare to their patients. Therefore, credibility in seeking health information refers to the reliability and authenticity of the source of the health information [63,64]. Based on the PT, there are several routes that NISs follow in assessing the credibility of health information. One way is to consider the authentication of the website. For example, health information from a well-respected medical organization (e.g., NICE, NHS, WHO, MoH) or peer-reviewed websites, which are acknowledged to be more reliable than websites, does not have similar authenticity.

Based on the PT, using professional organization websites had a median score of 0.5, which indicates that participants had negligible regard for these websites, and did not regard them as being authoritative and accurate enough to be used in daily clinical practice. Moreover, checking the domain name (.edu or .org) had a median score of 3, thus participants considered the domain name while seeking for health information in only three CSs out of eight as a strategy for checking the credibility of the resource.

Another method of assessing the quality of the information is to look for recent health resources, as more up-to-date peer-reviewed resources are considered to offer the most reliable evidence for EBP. However, the PT showed that NISs were less likely to check the date of the information they got when they were seeking health information. Only one out of eight clinical statements was located through checking that the information obtained was up-to-date. Similarly, a study by Raj et al. (2015) surveyed 100 health workers about seeking health information in their work; 20% agreed that outdated health information was considered a reason for not using health information in their work [65]. Outdated health information can lead to inefficient clinical decisions, such as using old guidelines, which in practical deployment can result in reduced QoC and even patient harm, such as delayed recovery or compromised patient safety [66].

Another consideration is the website’s reputation criteria, which had a median of 7, when assessing the reliability of the publication information; it scored only 3.5 with regard to the participants’ process of seeking health information. This finding is contrary to the outcomes of Komissarov and Murray (2016), who investigated factors that influenced 542 undergraduates’ ISBs and opportunities for EBI practice. They found that 43% of students rated author reputation for sources as “somewhat important”, and 32% rated it as “very important” for finding EBI; furthermore, only 1% rated the author and source reputation as “not important” [67]. Others have suggested that differences in access and resource types used may be influenced by a person’s specific biases and background knowledge [3,31].

The finding that nursing students prioritize access to accurate, reliable, and up-to-date health information has significant implications for clinical practice, policy, and future research. Ensuring that nursing students have access to reliable information sources is crucial for their education, requiring institutions to prioritize providing high-quality resources and teaching critical appraisal skills [68]. Policies and curricula may require updating in order to reflect the increasing reliance on digital resources in healthcare, necessitating training in information and digital health literacy [69]. Future research should explore students’ information needs and the effects of educational interventions on their ability to access and utilize health information effectively. However, limitations include the potential incompleteness of our findings and the limited generalizability to specific educational settings or student populations, highlighting the need for further research to address these gaps.

Guidance is needed to help NISs understand what authentic health information is, and how to identify the existing supports and services for finding it. Similarly, Willemse et al. (2019) said that healthcare institutions need to evaluate the health information sources that will be used in clinical practice [70]. In this regard, medical databases can provide a great benefit as regards accessing various health topics considering latest research studies, guidelines, and EBP recommendations [71,72]. According to Ryan (2016), nursing students who used medical databases to obtain health information mentioned increased ability to locate EBP recommendations, and utilized them in practice [73]. However, the PT indicated that the NISs’ were rarely able to integrate medical databases to find health information.

One significant finding from our study was the low scores related to using databases, comparing resources, and linking strategies among nursing students. This finding is particularly important as it indicates potential gaps in the information literacy skills of nursing students, which are essential for EBP and informed decision-making in clinical settings. Previous research has shown that proficiency in database searching and linking strategies is crucial for accessing and utilizing relevant evidence to inform nursing practice [74]. The lowest score for the PT pertained to “using medical databases”, with a median of 0.5. Thus, databases such as PubMed and MEDLINE are unlikely to be used for obtaining clinical answers among NISs. A large number of NISs fell below the average score, which could indicate that there are significant gaps in skill levels related to using medical databases. It was also found that NISs did not think that databases were authoritative and accurate enough to be used in daily clinical practice.

It was found that NISs still prefer to search for information on resources such as Google, rather than searching medical databases. Conversely, Zafar (2013) found that 38% of students used online databases, while 70% were keen to have special training for the use of databases [75]. Our study suggests that nursing students may not possess adequate skills in this area, highlighting the need for targeted interventions and educational initiatives to enhance their information literacy competencies. Addressing these gaps in information literacy among nursing students is imperative to ensure they are equipped with the necessary skills to navigate and critically appraise the vast amount of health information available in clinical practice.

Linking strategies refer to the methods employed to navigate relevant information among the enormous amount of online health information resources [76]. One of the linking strategies utilized by NISs involves using multiple resources, which had a median score of 3 (thus, five out of eight clinical statements were answered through using the first resource that contained the needed information). Seeking different resources has some disadvantages, and might lead to irrelevant resources. Maximized search results lead to long, undifferentiated lists of both appropriate and inappropriate information resources. Nurse participants who used Google or other search engines were, on average, willing to browse through eight documents before giving up, indicating a limited tolerance for extensive search efforts. Furthermore, participants were also unwilling to dig deeply into a resource, preferring to abandon it rather than explore multiple internal hyperlinks [77].

Another unanticipated finding of the PT was that “compare resources” had a median score of 2, indicating that participants are less likely to use strategies such as split-screen, find on page, or opening two windows. Furthermore, NISs discovered inconsistencies in health information resources (with a median of 2), which means that participants had experience of finding the resources that provided conflicting health information in two CSs out of eight. Nursing students need to use strategic thinking to compare and contrast the health information obtained and critically evaluate the sources of information, in order to find inconsistencies and critically evaluate the health information resources [8].

### 4.2. Engagement with Clinical Statements

Researchers have acknowledged that problem-solving approaches with a clear TA task may encourage respondents to identify problems with the task, and resulted in a more engaged approach [78,79]. In this research, NISs acknowledged that clinical statements were clear, and that this encouraged them to start the ISP. The findings indicate many positive aspects of the engagement with the clinical statements during the TA task: NISs successfully completed 112 statements out of a total of 112 statements (i.e., a 100% success rate). Also, NISs mentioned words to express their thoughts on clinical statements, such as “interesting”, “doubting”, or “curious”, as indicators of their impression. Moreover, as discussed in the interpretation of results, NISs were able to initiate the process of seeking health information without asking for clarification. These results provide some evidence that clinical statements captured the participants’ attention and would give an accurate reflection of the ISP.

Research suggests that young nurses and nursing students with a lack of experience find it difficult to use various search skills, functions, and features when seeking information pertaining to different nursing topics in practice [80]. Consistent with this, NISs’ median performance score for seeking health information based on the clinical statements in this study ranged between 7 and 9. Participants had an average score of 8 out of 17 in relation to applying quality assessment in seeking health information for each clinical statement. The highest clinical statement score was 9 for CS5, in which the NISs sought relevant information to address daily clinical practice in the critical care unit related to the recent pandemic, which regards patient position in ARDS. This score could be influenced by the spread of COVID-19 topics and care in the field of clinical care.

The second highest clinical statement score was 8.5 for CS8, relating to NISs seeking relevant information to educate patients about methods of breathing, followed by a score of 8 for three clinical statements, CS2, CS3, and CS7, concerning attention to understanding nasogastric tube contraindication, GCS, and medication indication (respectively). The two lowest scores were 7.5 and 7 for CS4 and CS6 (respectively), which show that NISs might not find it easy to understand the medical abbreviations of ECMO or obtain information related to pain management, or that it may be difficult to apply a quality search due to indirect clinical statements. The clinical statement in this regard was “No laboratory test can determine the presence or severity of pain”. Overall, by using the PT, clinical instructors can identify nursing topics where further training is needed due to NISs’ difficulty in obtaining EBHI, such as medication indications.

### 4.3. Challenges and Future Research Directions

Overall, creating a tool to assess the skills of nursing students in seeking EBHI presented multifaceted challenges. Firstly, delineating the parameters of EBHI skills was complex, as it entailed identifying and prioritizing competencies crucial for nursing practice in an era of evolving healthcare landscapes. TA tasks reflected the participants’ activities regarding clinical statements that represented a typical nursing care encounter and how a participant interacted during the online session. Balancing the need for breadth and depth in content coverage was pivotal, so as to ensure that the tool comprehensively assessed students’ abilities to locate, evaluate, and apply evidence in clinical decision-making. Additionally, crafting items that accurately reflect the nuanced nature of EBHI skills while remaining clear and concise for student comprehension posed a considerable challenge, requiring iterative refinement and validation processes.

Developing a tool to evaluate the EBHI skills of nursing students entailed navigating several intricate challenges. One such obstacle was striking a balance between comprehensiveness and practicality in item selection. Ensuring that the tool covered a wide range of EBHI competencies while remaining concise and user-friendly demanded careful deliberation and iterative refinement. Additionally, aligning the tool with established frameworks and standards for EBHI represented another layer of complexity, requiring meticulous attention to detail and validation against existing measures. Moreover, adapting the tool to accommodate the evolving landscape of healthcare information and technology presented ongoing challenges, necessitating regular updates and revisions to maintain relevance and effectiveness. Addressing these challenges called for a multidisciplinary approach, drawing on expertise from nursing education, health informatics, and psychometrics to develop a robust and adaptable assessment tool capable of meeting the diverse needs of nursing students in the digital age.

Future research could explore the effectiveness of different educational strategies in improving information literacy skills among nursing students, and evaluate their impacts on clinical decision-making and patient outcomes. However, it is essential to acknowledge the limitations of our study, such as the specific context and sample characteristics, which may influence the generalizability of the findings. Further research is needed to confirm and expand upon these results in diverse nursing education.

## 5. Conclusions

The process of developing the tool for evaluating EBHI skills among nursing students was fraught with challenges, but was ultimately rewarding. The iterative process of refining the tool in response to feedback and emerging research not only enhanced its validity and reliability, but also underscored the importance of collaboration and adaptability in tool development. By addressing the complexities inherent in assessing EBHI skills, this tool holds promise for empowering nursing students with the competencies needed to navigate the ever-expanding realm of healthcare information effectively. As we move forward, continued engagement with stakeholders, ongoing validation efforts, and proactive updates will be essential to ensure that the tool remains robust and reflective of current best practices in EBHI assessment. Through these efforts, we can better equip the next generation of nurses to critically evaluate, integrate, and apply EBHI in their practice, ultimately improving patient outcomes and advancing the field of nursing.

This study sheds light on the challenges faced by NISs in information retrieval, particularly regarding search behavior. The findings provide valuable insights into the limitations of current ISP among NISs, and underscore the need for targeted interventions to enhance their information literacy skills. If these challenges are addressed by nursing educators, curriculum designers, and preceptors, nursing students can improve their ability to access and utilize relevant evidence in clinical practice, ultimately contributing to improved practice outcomes such as critical thinking. Moving forward, future research should focus on developing and evaluating effective educational interventions to support nurses in navigating digital information resources more effectively, thus bridging the gap between information needs and retrieval capabilities in healthcare settings.

The performance tool can be used as a valuable tool for researchers, educators, or clinical instructors to understand NISs’ decision-making process, and to better prepare for providing high-quality patient care. Overall, there is a need to consider a feedback mechanism in the PT, via a self-reflection based on self-assessment, evaluation by a faculty member, peer review, or a combination of these methods. Feedback on NISs’ performance should be given in a timely and constructive manner, so as to help them to identify areas for improvement and track their progress in the clinical placement. By assessing NISs’ performance and providing feedback, clinical instructors can support NISs’ professional development and maintain patient safety.

## Figures and Tables

**Table 1:** Clinical statements.

N	Focus	Clinical Statements
1	Sepsis	Serum lactate is an important indicator of the septic patient’s prognosis, with mortality dropping significantly as the lactate level decreases.
2	NGT contraindication	A nasogastric tube is considered as a contraindication in a patient with a basal skull fracture.
3	Glasgow coma scale (GCS)	Patients with GCS lower than 8 require urgent airway and breathing management.
4	Extra Corporeal Membrane Oxygenation (ECMO)	ECMO is a support not a treatment to provide stability while the underlying cause is treated.
5	Patient position	Prone positioning might improve overall survival in severe ARDS.
6	Pain management	No laboratory test can determine the presence or severity of pain.
7	Medication indication	Heparin reduces pulmonary compromise and intravascular coagulation in fat embolism patients.
8	Patient education	A patient asked about the purpose of the pursed-lip method of breathing.

**Table 2:** Yes/no questions derived from criteria emerging from the collected TA data.

No.	Question	Criteria	Focus
1	Does the NIS correctly interpret the clinical statements?	Interpretation	There was no need to ask the researchers to clarify the clinical statements.
2	Does the NIS use appropriate keywords while seeking health information?	Using keywords	NIS used keywords to expand and enhance their search results.
3	Is the NIS able to use the search engine properly?	Search engine properly	NIS was able to transform a clinical question or a need for health information into a searchable sentence.
4	Are the sources sought by NIS relevant to address the clinical statement?	Relevant	NIS was able to find resources related to the clinical statement.
5	Does the NIS search mainly professional health information?	Authorized	NIS used professional organization websites (NICE, NHS, WHO, MoH) or hospital websites.
6	Does the NIS use their prior knowledge to construct meaning?	Prior knowledge	NIS was able to use his/her prior knowledge to construct the meaning.
7	Does the NIS seek clarification of the meaning when they detect unknown words, abbreviations or medical terminology?	Seek clarification	When the NIS detected unknown words while seeking health information, he/she sought meaning to satisfy the health information needs.
8	Does the NIS check the domain name? (.edu, .gov)	Check domain name	NIS was looking for websites with.edu or.org domains.
9	Does the NIS use the medical databases?	Medical databases	NIS was able to integrate databases where medical information is stored, such as CINAHL or PubMed.
10	Does the NIS question the website’s (organization) reputation in terms of health information?	Website reputation	When the NIS questioned if the source is able to be trusted, such as Wikipedia.
11	Does the NIS assess the author information?	Assess author information	When the NIS assessed the reliability of the author (publication information).
12	Does the NIS seek the published or posted date?	Up-to-date	NIS was looking for the current health information.
13	Does the NIS match the usefulness of the information found to the specific needs of the clinical question posed?	Usefulness	When the NIS examined the usefulness of health information in clinical practice. (clinical judgment) (using pictures, video)
14	Does the NIS use more than one source of information?	More than one resource	When the NIS used more than one resource to obtain the health information.
15	Does the NIS use strategies to compare and contrast the information from different sources?	Compare resources	When the NIS used strategies that help to choose a resource, such as split screen to compare resources or information.
16	Does the NIS weight the value of different information sources based on the quality of the difference sources?	Valuation	When the NIS detects inconsistency or evidence relationships in health information websites.
17	Does the NIS satisfy with information obtained?	Satisfaction	When the NIS completed the search process and was satisfied with the information obtained.

**Table 3:** Participant scores in the TA sessions.

No.	Assessment Criteria																	
	Interpretation	Using Keywords	Search Engine Properly	Relevant	Authorized	Prior Knowledge	Seek Clarification	Check Domain Name	Medical Databases	Website Reputation	Assess Author Information	Up-to-Date	Usefulness	Multiple Resources	Compare Resources	Valuation	Satisfaction	Total
1	Y	Y	Y	Y	N	N	N	Y	N	Y	Y	N	Y	Y	Y	Y	Y	12
2	Y	Y	Y	Y	Y	Y	N	N	N	Y	N	N	Y	Y	Y	Y	Y	12
3	Y	Y	N	Y	Y	Y	N	Y	N	N	N	N	Y	Y	Y	N	Y	10
4	N	Y	N	Y	Y	N	Y	Y	N	Y	N	N	Y	Y	N	Y	Y	10
5	Y	Y	N	Y	Y	Y	N	N	N	Y	Y	N	Y	Y	Y	Y	N	11
6	Y	Y	Y	Y	N	N	Y	N	N	Y	Y	N	N	Y	Y	Y	N	10
7	Y	N	Y	Y	Y	Y	N	N	N	Y	Y	N	Y	Y	N	N	N	9
8	N	Y	Y	Y	Y	N	Y	Y	N	Y	Y	N	Y	Y	Y	Y	Y	13
Total	6	7	5	8	6	4	3	4	0	7	5	0	7	8	6	6	5	87 *

No. Key (Clinical Statements); Serum lactate is an important indicator of the septic patient’s prognosis, with mortality dropping significantly as the lactate level decreases. A nasogastric tube is considered as a contraindication in a patient with a basal skull fractures. Patients with GCS lower than 8 require urgent airway and breathing management. ECMO is a support not a treatment to provide stability while the underlying cause is treated. Prone positioning might improve overall survival in severe ARDS. No laboratory test can determine the presence or severity of pain. Heparin reduces pulmonary compromise and intravascular coagulation in fat embolism patients. A patient asked about the purpose of the pursed-lip method of breathing. * Total individual performance.

**Table 4:** The total performance scores of each participant and criteria.

No.	Assessment Criteria (AC)	NISs														** Total
		P1	P2	P3	P4	P5	P6	P7	P8	P9	P10	P11	P12	P13	P14	
1	Interpretation	* 6	8	6	8	7	8	8	7	6	5	7	7	8	2	93
2	Using keywords	7	8	7	8	7	4	7	8	7	7	8	6	8	6	98
3	Search engine properly	5	7	4	5	2	5	5	3	4	3	5	7	3	4	62
4	Relevant	8	6	8	8	7	8	7	8	8	8	8	7	7	8	106
5	Authorized	6	3	0	0	1	0	0	1	0	1	3	1	0	0	16
6	Prior knowledge	4	6	2	1	2	3	3	5	2	3	5	3	2	0	41
7	Seek clarification	3	4	3	2	1	1	1	3	3	2	0	4	1	1	29
8	Check domain name	4	2	6	6	1	5	0	3	2	6	1	0	4	3	43
9	Medical databases	0	0	1	0	3	3	0	3	0	0	1	2	0	2	15
10	Website reputation	7	3	6	8	7	5	3	8	7	6	8	7	8	6	90
11	Assess author info	5	2	0	7	1	2	4	3	2	5	5	4	5	2	47
12	Up-to-date	0	0	0	1	0	0	2	1	0	3	3	2	4	1	17
13	Usefulness	7	7	2	4	3	3	4	6	4	3	4	4	5	3	59
14	Multiple resources	8	5	1	7	0	1	4	4	1	3	3	6	1	1	45
15	Compare resources	6	3	0	6	0	1	4	4	0	3	3	5	0	1	36
16	Valuation	6	2	1	6	0	0	2	4	1	2	3	5	0	0	32
17	Satisfaction	5	8	5	6	8	8	8	8	8	7	8	8	6	5	98
*** Total		87	74	52	83	50	57	62	79	55	67	75	78	62	45	

* P1 was able to understand 6 out of 8 clinical statements. ** Total each criteria scores performance. *** Total each participants scores performance.

**Table 5:** The total performance scores of each participant under each clinical statement.

No.	CSs	NISs														** Total
		P1	P2	P3	P4	P5	P6	P7	P8	P9	P10	P11	P12	P13	P14	
1	Sepsis diagnosis	* 12	10	6	10	7	7	6	9	6	7	8	9	8	5	110
2	NGT contraindication	12	10	6	7	8	8	7	9	8	9	11	12	8	7	122
3	GCS	10	7	6	10	5	8	6	12	7	12	8	12	9	6	116
4	ECMO	10	12	6	12	6	6	7	11	7	8	13	8	7	6	117
5	Patient position	11	11	7	10	7	6	10	10	7	7	11	11	8	4	122
6	Pain management	10	8	5	11	6	7	7	9	6	7	8	7	7	5	101
7	Medication indication	9	8	7	9	5	9	10	9	8	7	8	9	7	7	112
8	Patient education	13	8	9	12	6	6	9	10	6	10	8	10	8	5	117
*** Total		87	74	52	83	50	57	62	79	55	67	75	78	62	45	

* P1 was able to score 12 out of 17 assessment criteria. ** Total of each clinical statement score’s performance. *** Total of each participant’s score performance.

## Data Availability

Data are contained within the article.
